# Entecavir Therapy in Turkish Adult Patients with Chronic Hepatitis B: One-Year Results from Izmir Province, Turkey

**Published:** 2010-09-01

**Authors:** Sukran Kose, Melda Turken Ulusoy, Gulgun Akkoclu, Ayhan Gozaydin

**Affiliations:** 1 Tepecik Research and Training Hospital, Clinic of Clinical Microbiology and Infectious Diseases, Tepecik, Izmir, Turkey

**Keywords:** Hepatitis B, Treatment, Entecavir

## Abstract

**Background:**

In the present study, we aimed to present the initial results of chronic hepatitis B patients who received entecavir (ETV) therapy in our hospital in Izmir, Turkey.

**Methods:**

A total of 52 patients were enrolled in the study. ETV was given in a dosage of 0.5 mg/day and 1 mg/day to 50 patients without Lamivudine/Adefovir (LAM/ADV) resistance and to 2 patients with LAM resistance, respectively. ETV was given in a dose of 0.5mg/day every three days to one patient with a renal transplant. The treatment duration was 48 weeks.

**Results:**

Out of a total of 52 patients, 23 (44.23%) were hepatitis B e antigen (HBeAg)-positive, and 29 (55.77%) of them were HBeAg-negative. In 29 HBeAg-negative patients, early biochemical and virological responses were 82.6% and 100%, respectively. These responses were 97% and 79.3% in the 12th month. In HBeAg-positive patients, early biochemical and virological responses were found to be 78.3% and 82.6%, respectively. They were 100% and 52.2% in the 12th month. HBeAg s  oconversion developed in 4.5% of HBeAg-positive patients.

**Conclusions:**

According to our one-year ETV treatment results, both HBeAg-negative and -positive patients had high biochemical and virological response rates. Their HBeAg seroconversion rate was 4.5%. In conclusion, more studies of longer duration are needed to understand the required duration of treatment, to assess its long-term effectiveness, and to check the resistance and side effects of ETV. There is also a need to have late-phase results after treatment.

## Introduction

Hepatitis B virus (HBV) affects more than 2 billion individuals worldwide, and approximately 350 million people are long-term HBV carriers [1]. Turkey has medium endemicity for incidence of HBV infection, and the estimated number of HBV carriers is 2.4-6 million people [2]. Chronic hepatitis B (CHB) is induced by the chronic replication of HBV in the liver and has a poor prognosis, with 20–40% of infected individuals developing liver cirrhosis, noncompensated liver disorder, or hepatocellular carcinoma (HCC) [3]. Studies on hepatitis B seroprevalence were done in different regions of Turkey. Kangin et al. carried out a large-scale study in endemic regions and found that the seroprevalence was 8.1% among 10,391 children [4].In northern Turkey, the seroprevalence of hepatitis B among pregnant women was found to be 2.1% [5]. As is well-known, hepatitis B is very prominent among blood donors, and the general seroprevalence ratio was found to be 4.19% in a previous study carried out by Emekdas et al.[6]. Treatment  of CHB is aimed at the sustained inhibition of HBV replication and at the remission of liver disease [7], ultimately preventing progression to liver cirrhosis or HCC [8]. For its treatment, agents like interferon alpha (IFN a), pegylated interferons (PEG-IFN), lamivudine (LAM), adefovir (ADV), entecavir (ETV) and tenofovir (TDF) have been used in many countries, as well as in Turkey. The antiviral agents ETV and TDF were introduced to Turkey in 2007 and 2008, respectively. ETV is a strong and selective inhibitor of HBV DNA polymerase. Previous studies have shown that its reliability and its side-effect profile are similar to that of LAM. In our study, we assessed the shortand long-term effects of ETV on chronic hepatitis B patients receiving ETV treatment, and they are followed at three-month intervals.

## Materials and Methods

Routinely, the naïve or previously treated hepatitis B patients have been followed in our outpatient clinic and their alanine aminotransferase/ aspartate aminotransferase (AST/ALT), hepatitis B surface antigen (HBsAg), antibody to hepatitis B surface antigen (anti-HBs), hepatitis B e antigen (HBeAg), antibodies to hepatits B e antigen (anti- HBe), hepatitis B virus DNA (HBV-DNA)levels are checked at three-month intervals (in the 3rd, 6th , 9th and 12th month). Additionally, LAM- and ADVresistance tests were applied to all individual subjects. When the subjects first began to be followed, liver biopsies were done on them.

The following criteria were used for enrolling the patients in the study: elevated levels in liver function tests, elevated levels of HBV DNA (> 105 copy/ml in HBeAg positive patients, >104 copy/ml in HBe negative patients), and histology activity index (HAI) score of =4 and fibrosis =2 in both groups.

Patients having HIV infections were excluded from the study because of the contraindications   A total of 52 patients were enrolled in the study. A dose of 0.5 mg/day and 1 mg/day of ETV was given to 50 patients without LAM/ADV resistance and to 2 patients with LAM resistance, respectively. The drug was administered in a dose of 0.5mg/day, every three days, to one patient with a renal transplant. The treatment duration was 48 weeks.

The resistance against LAM and ADV was evaluated using the multiplex PCR and reverse hybridization tools (INNO-LIPA HBV DR v2, Innogenetics, Belgium). According to the criteria of the Turkish Association for the Study of the Liver, an ALT/AST normalization in the 3rd, 6th and 12th month is accepted as a biochemical response (BR) in chronic hepatitis B patients, and 2 logarithmic decreases in the HBV DNA level in the 3rd month, lower than 104 copy/ml in the 6th month and an undetectable level in the 12th month are accepted as a virological response (VR). In HBeAg-positive patients, the detection of anti-HBe antibodies is accepted as a serological response (SR).

## Results

Nineteen (36.6%) and 33 (63.4%) of the patients were female and male, respectively. Their mean age was 37.9 (between 17 and 67) years. Thirtyeight (73.1%) of the patients were naïve, while 14 (26.9%) patients had previous nucleos(t)ide analogue treatment (with LAM and/or ADV). LAM and ADV resistance were detected in two patients. Out of 52 patients, 23 (44.2%) were HBeAgpositive, while 29 (55.8%) were HBeAg-negative. ETV treatment results and the patients’ details are provided in Table 1 and Table 2. HBeAg seroconversion developed in 4.5% of the HBeAgpositive patients. The analyses showed that there is no significant relationship between the previous nucleoside use and the virological response; between the level of baseline HBV DNA and virological response; between the beginning level of fibrosis value and virological response in any of the groups (data not shown).

**Table 1:** ETV Treatment results of HBeAg positive and HBeAg negative patients.

	**HBeAg (+)**	**HBeAg (-)**
Number (%)	23 (44.23%)	29 (55.77%)
Mean age	37.8	37.4
Male	15 (65.2)	18 (62.1%)
Female	8 (34.8)	11 (37.9%)
Naive	18 (78.3%)	20 (69.0%)
Previously treated	5 (21.7%)	9 (31.0%)
LAM/ADV resistance	0	2
ALT	110.12	111.8
HBV DNA	5x107 k/ml	6.3x107 k/ml
Histologic Activity Index	8.84	8.82
Biological Response (%)		
3rd montd	78.3	82.6
6td montd	87.0	97.0
12td montd	100.0	97.0
Virological Response (%)		
3rd montd	82.6	100.0
6td montd	60.9	97.0
12td montd	52.2	79.3
Serological Response (%)		
12td montd	34.8	-

**Table 2:** The response rates of the patients to ETV treatment

		**3^rd^ month**		**6^th^ month**		**12^th^ month**		
		**BR^a^**	**VR^a^**	**BR**	**VR**	**BR**	**VR**	**SR^a^**
Naïve (n= 38)	HBeAg (+) (n=18)	72.2 (13/18)	83.3 (15/18)	83.3 (15/18)	66.7 (12/18)	100(18/18)	55.6 (10/18)	38.9 (7/18)
	HBeAg (-) (n= 20)	80 (16/20)	100 (20/20)	95.0 (19/20)	95.0 (19/20)	95.0 (19/20)	80 (16/20)	
								-
	HBeAg (+) (n= 5)	100 (5/5)	80 (4/5)	100 (5/5)	40 (2/5)	100 (5/5)	40 (2/5)	20.0 (1/5)
NA aexperienced (n= 14)								
						
	HBeAg (-) (n= 9)	88.9 (8/9)	100 (9/9)	100 (9/9)	100 (9/9)	100 (9/9)	77.8 (7/9)	-

^a^ BR: Biochemical response; VR: Virological response; SR: Serological response; NA: Nucleoside analogues

## Discussion

In the present study, the results of the 48-week analysis of this study population of 38 nucleosidenaïve and 14 non-naïve Turkish patients with chronic hepatitis B were presented. A resurgence of viral replication after the specific treatment, and drug resistance, are two common problems in the antiviral therapy of CHB patients. Previous studies have shown that ETV has a high genetic barrier, with a low incidence of resistance, in nucleoside-naïve CHB patients. The cumulative probability of genotypic resistance to ETV remains low, at the ratio of 1.2% after 6 years of therapy, for nucleoside-naïve patients [9]. Other nucleoside or nucleotide analogues such as LAM or ADV have higher reported rates of resistance – up to 65% with LAM and 29% with ADV within 5 years [7][10]. In chronic hepatitis B patients with LAM resistance, the virological response was found to be 59.6% after ADV treatment [11].

In the study carried out by Yenice et al., after five years of LAM therapy, the virological response was found to be  36.4% and 80% in HBeAg-positive and HBeAg-negative patients, respectively [12]. Additionally, in another five-year study, four different therapies were applied to chronic hepatitis B patients, and their virological response was found to be 55%, 42%, 96% and 94% with LAM, ADV, ETV and TDF, respectively [13]. In our study, at the end of one year of ETV therapy, the virological response was found to be 52.2% and 79.3% in HBeAg-positive and HBeAg-negative patients, respectively.

Longer treatment is needed to maintain sustainable viral suppression, especially in HBeAg-negative CHB patients. Thus, potential antiviral drugs with a good resistance profile over a long-term treatment period are recommended [14]. Although PEG-IFN alpha can be another option for suppressing viral load, it is known that long-term usage of PEG-IFN alpha is undesirable because of adverse events.

In the previous studies conducted on naïve CHB patients, different results were reported. Hadziyannis et al. have reported that after one year of t  thrapy, the virological and biochemical responses were 90% and 78%, and 67% and 68%, in HBeAg-negative and HBeAg-positive patients, respectively [10]. Chang etal. [15] compared ETV and LAM treatment of naïve HBeAg-positive CHB patients and after the first year of the study, the virological response was found to be 67% in the ETV group and 36% in the LAM group. Biochemical response rates were 68% and 60% in the ETV and LAM groups, respectively. Lai et al. compared ETV and LAM treatment of naïve HBeAgnegative CHB patients; they have found virological responses of 90% and 72% in the ETV and LAM groups, respectively. The biochemical response of the ETV group was found to be 78% while that of the LAM group was found to be 71% [16].

In our study, after a 48-week treatment with ETV, the virological and biochemical response rates of naïve CHB patients were higher in HBeAgnegative patients (79.3% and 97.0%) than in HBeAg-positive patients (52.2% and 100%). The virological response rates of naïve HBeAg-negative patients   (80%) and HBeAg-positive CHB patients (55.6%) were found to be lower than in the other studies; however, the biochemical response rates in both groups (100% in HBeAg-positive naïve CHB patients, and 95% in HBeAg-negative naïve CHB patients, at year one of ETV therapy) were found to be higher when compared to the results of other studies. The reason for these differences is doubtless due to the smaller number of patients studied. We need to observe a large number of CHB patients over a longer period. A total of 14 patients (5 HBeAgpositive, 9 HBeAg-negative) had previous treatment with other nucleoside analogues. Both the virological and the biochemical response rates of these patients in the first year of ETV therapy were found to be higher than the ones mentioned in other studies (Table 2).

Studies have shown that in LAM-resistant CHB patients, response rates to ETV over a longterm treatment period should decrease, because of the probability of cross resistance between the two drugs. In the study carried out by Hadziyannis et al. [10], virological response rates to ETV in naïve CHB patients at year three were 94%, whereas these response rates for the LAM-resistant group were 40%. In the same study, naïve patients who had received three years of ETV treatment had <1% resistance; however the resistance rate became 30% after three years of ETV treatment, in the LAM-resistant group. Nagasaki et al. [17] have reported that in 3 out of 4 LAM-resistant subjects, resistance developed in the period from the 52nd to the 130th week. In the study conducted by Tilmann [18] in Germany, 9% of the subjects with LAM resistance developed resistance after 2 years. In a multi-centered study involving many LAM-resistant HBeAg-positive CHB patients, the group that continued LAM treatment for 52 weeks had a 4% virological and biochemical response (6/145), but this rate was 55% in the group which was treated with 1 mg/day of ETV. A total of 10 out of 141 patients showed genotypical ETV resistance and 2 had virological rebound. In our study  , 2 patients who had genotypical LAM resistance at baseline responded to 1 year of ETV treatment, however no genotypical resistance tests were conducted in the present study.

In Turkey, it is reported that multidrug resistance against ADV and ETV was also detected in LAMresistant hepatitis B patients who received LAM therapy for one year [19].

It is reported that extended therapy with ETV over 5 years maintained or increased rates of HBV DNA suppression and ALT normalization. Additional patients also achieved HBeAg loss and seroconversion. ETV provides sustained viral suppression with minimal resistance during longterm treatment of HBeAg-positive CHB [20]. In our study, after one year of treatment with ETV, the response rates of HBeAg-negative patients were found to be elevated. HBeAg-positive patients’ virological and biochemical response rates were also found to be high at the end of the first year, although the HBeAg seroconversion rate was 4.5%. In conclusion, we need to observe more patients for a longer  period of time, in order to assess the long-term effectiveness, safety and resistance profile of ETV.
